# Overcoming Language Barriers in Paramedic Care With an App Designed to Improve Communication With Foreign-Language Patients: Nonrandomized Controlled Pilot Study

**DOI:** 10.2196/43255

**Published:** 2023-03-23

**Authors:** Frank Müller, Dominik Schröder, Eva Maria Noack

**Affiliations:** 1 Department of General Practice University Medical Center Göttingen Göttingen Germany

**Keywords:** app, emergency medical technicians, language barriers, limited language proficiency, migrant, paramedic, prehospital emergency care, refugee, translator

## Abstract

**Background:**

Communication across language barriers is a particular challenge for health care providers. In emergency medical services, interpreters are mostly not available on rescue scenes, which jeopardizes safe and high-quality medical care. In a cocreative process together with paramedics and software designers, we developed a fixed-phrase translation app with 600 phrases and 18 supported languages that supports paramedics when providing care to foreign-language patients. This paper reports on the results of a pilot study to evaluate the app’s effect on paramedic-patient communication.

**Objective:**

This study aims to gain insights into the efficacy and feasibility of a multilingual app that helps paramedics to communicate with patients who are not proficient in the local language.

**Methods:**

A 3-armed nonrandomized interventional pilot study was conducted in 4 rescue stations in the German Federal State of Lower Saxony: 3 rural areas and 1 in urban environment. The intervention group comprised rescue missions with patients with limited German language proficiency (LGP) with whom the app was used; control group 1 comprised LGP patients without app usage; and control group 2 consisted of rescue missions with German-speaking patients. For each rescue operation with LGP patients, paramedics filled out questionnaires about the communications with patients. From standardized Rescue Service Case Protocols, we extracted information on patient demographics (age and sex), clinical aspects (preliminary diagnosis and Glasgow Coma Scale), and rescue operation characteristics (time spent on emergency scene and additional dispatch of emergency physicians). The primary outcome was the paramedics’ perceived quality of communication with LGP patients. The secondary outcome was the ability to obtain necessary information from patients and the ability to provide important information to patients. A linear regression model was applied to assess the impact of the app on perceived communication, controlling demographic factors, and severity of illness.

**Results:**

A total of 22 LGP patients were recruited into the intervention group and 122 into control group 1. The control group 2 included 27,212 German-speaking patients. LGP patients were more than 2 decades younger than German-speaking patients. App usage among LGP patients was associated with higher perceived overall quality of communication (0.7 points on a 5-point Likert scale, *P*=.03). Applying a linear regression model controlling for age, sex, and Glasgow Coma Scale, the quality of communication was associated with an increase of 0.9 points (95% CI 0.2-1.6, *P*=.01). Compared to either German-speaking patients or LGP patients, paramedics spent 6-7 minutes longer on an emergency scene when the app was used (*P*=.24).

**Conclusions:**

The use of the app suggests a relevant improvement in communication with patients with limited proficiency in the locally spoken language in paramedic care. The small sample size and the lack of randomization reduce the generalizability of the findings.

**Trial Registration:**

German Clinical Trials Register DRKS00016719; https://drks.de/search/de/trial/DRKS00016719

## Introduction

Medical professionals in the Western world are increasingly providing care to patients with limited proficiency in the locally spoken language [1,2]. In recent decades, the linguistic heterogeneity of people living in Germany has increased. Between 2015 and 2020, more than 2.3 million refugees sought protection in Germany [3,4]. The war in Ukraine resulted in the reception of another 900,000 people [5]. Germany is also a relevant destination for employees from all over Europe and for tourists and business travelers from all over the world [6].

Trained interpreters can be considered the gold standard to gap language discordance. While access to interpretation services can be prearranged in hospital or ambulatory care, professional interpreters are most often not available in prehospital emergency settings where care is provided by emergency medical services (EMS). Paramedics often rely on the interpretation skills of bystanders or try to communicate in a third language, for example, English [7,8]. Ad hoc translators, such as Google Translate, are still not considered sufficiently accurate for translational services in health settings [9,10], especially in lesser used languages [11]. Moreover, such services often require reliable network coverage, which is rarely guaranteed in rural areas in Germany.

Misunderstandings in the medical history and misinterpretation of symptoms can lead to severe errors and jeopardizes the safety and quality of provided emergency treatment [12-14]. Research has shown that lack of language interpretation leads to ineffective use of resources [15] and can cause delays in the delivery of care [16-18]. Additionally, language discordance has been shown to be a barrier to the use of EMS [19], and it has been reported that foreign language–speaking patients call EMS for other reasons than nonforeigners [20]. In an action-oriented participatory approach, we developed together with paramedics and software designers an app that supports paramedics when providing care to foreign-language patients [21]. We assessed paramedics’ perception and evaluation of the usability of the app [22]. This paper reports on the results of a nonrandomized controlled pilot study using this app for the first time in a real-life setting, evaluating the paramedic-rated communication with foreign-language patients [23].

## Methods

### Overview

This study was conducted as a nonrandomized controlled pilot study to explore general feasibility and assess the app’s effect on communication with non–German-speaking patients. The intervention group consisted of patients with limited German language proficiency (LGP) defined as an existing communication barrier, with whom the app was used. The following two control groups were recruited: control group 1 comprised LGP patients with whom the app was not used, and control group 2 comprised German-speaking patients and serves as baseline to allow comparison with regard to medical and rescue operations characteristics as they may differ to LGP patients [20]. As details on the study procedures are outlined in a separate study protocol [23], we will revisit the study only briefly.

### Intervention

**Figure 1:**
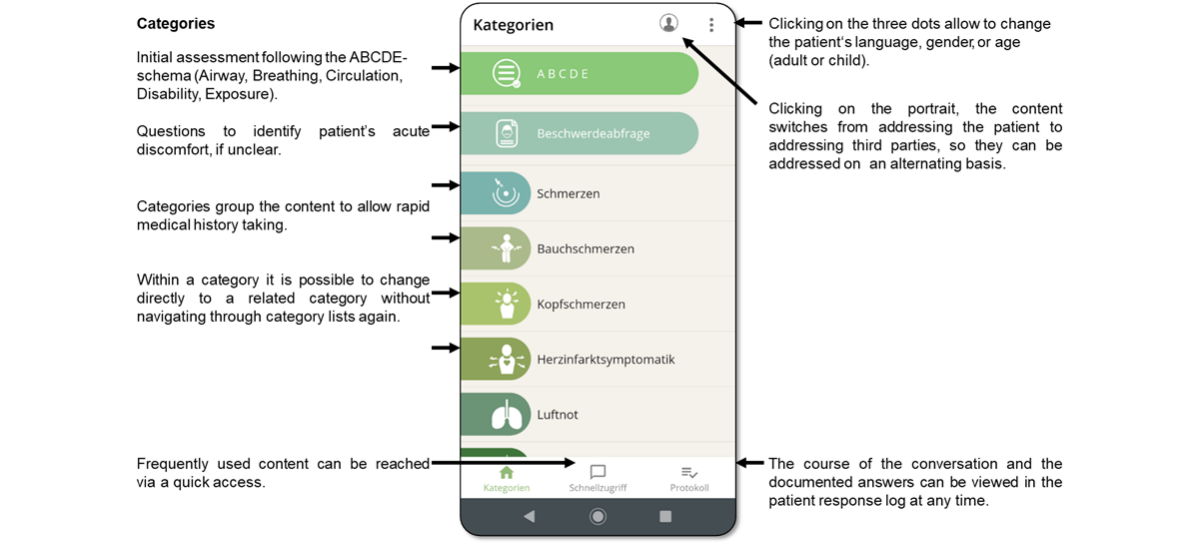
Screenshot of the category screen and explanation of some of the app’s functions.

**Figure 2:**
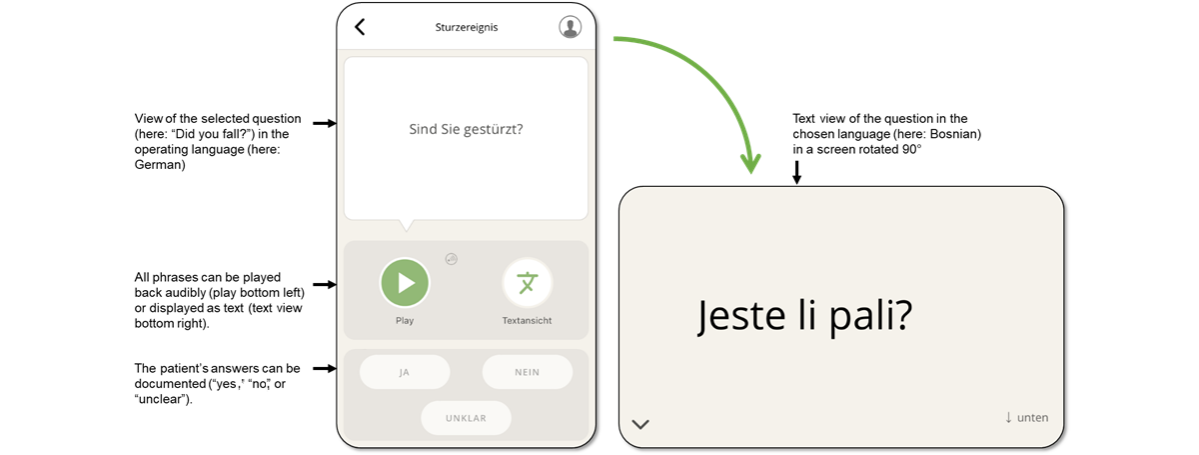
Screenshot of a screen showing how a selected sentence can be played back or displayed as text.

The developed app assists paramedics to overcome language barriers when providing care to foreign-language patients. It is suitably adapted to the specific circumstances of rescue operations, with offline usage capabilities, and maintains data confidentiality. The app is operated by the paramedics and enables them to ask questions and provide information about examinations or measures taken by paramedics. The app works as a fixed-phrase translator. In each language, the app contains 600 standard phrases that are, depending on the supported language, tailored to consider the gender and age of the person seeking help. Thus, adult and pediatric patients are addressed with appropriate wording, as well as third parties, such as relatives or parents of sick children. The content is grouped in categories with recognizable icons, to allow rapid medical history taking adapted to different disorders and call reasons respectively. There are categories for physical examination, informative and reassuring sentences, questions concerning preexisting conditions, drugs, intolerances, and patient documents. Within categories, the content is clustered according to the paramedic approach of structuring a rescue mission. Figure 1 includes a screenshot of the category screen and the app’s main functions. The app’s functionalities and navigation take into account the variety and complexity of rescue missions. All phrases can be displayed as text or playback audio using the loudspeaker of the cellphone (see Figure 2). The course of the app-based communication is automatically saved in a log; patients’ responses, for example, “yes” or “no,” or localization of pain on a figure can be logged by paramedics. If done so, the log resembles a chat history, showing the complete course of conversation. Alternatively, the information received can be presented in a structured way using the SAMPLE history scheme (assessment used in prehospital emergency care including Symptoms, Allergies, Medications, Past medical history, Last oral intake, Events prior to incident). For this study, the app is run on a Motorola Play Z2 Android smartphone with external speakers to be of use in noisy environments.

All phrases were translated and audio-recorded by professional interpreters. In this study, the app supported the following 18 languages: Arabic, Bosnian, Croatian, Czech, Dari (Persian), English, Farsi (Persian), French, German, Italian, Kurdish-Sorani, Lithuanian, Pashto (Afghani), Polish, Russian, Serbian, Spanish, and Turkish.

### Hypothesis

The primary hypothesis was that the use of the app improves the quality of communication with LGP patients as perceived by paramedics. A further hypothesis was that the use of the app improves the ability of paramedics to obtain relevant information from LGP patients as well as the ability to provide LGP patients with necessary information. Exploratorily, it was observed if the use of the app affects the time paramedics spent on the emergency scene. Increased on-scene time could indicate that the app may delay the transport to the hospital and is therefore considered an important factor.

### Measures

Collected variables comprised patient demographics (age and sex), clinical aspects (preliminary diagnosis and Glasgow Coma Scale [GCS]), and rescue operation characteristics (time spent on emergency scene and additional dispatch of emergency physicians), which were extracted from deidentified electronic and paper-based standardized Rescue Service Case Protocols. Paramedic-rated communication (primary outcome) and quality of gathered information (secondary outcome) were derived from a paper-pencil questionnaire that was filled out by paramedics for each rescue operation with LGP patient, that is, intervention group and control group 1. The items assessing communication, including the quality of the content of the conversation, were rated on a 5-point Likert scale (Table 1) [24]. Free-text entries were included to specify which information could not be obtained from or given to patients.

**Table 1:** Perceived quality of communication with and without the app.

			Intervention group				Control group 1			*P* value^a^		
			Participants, n (%)		Mean (SD)		Participants, n (%)		Mean (SD)			
**Item 1: “The overall communication with the patient was…”**					2.8 (1.1)				3.5 (1.1)	.03		
	(1) Very easy	3 (17.6)				3 (2.7)						
	(2)	2 (11.8)				16 (14.3)						
	(3)	7 (41.2)				41 (36.6)						
	(4)	5 (29.4)				21 (18.8)						
	(5) Very difficult	0 (0)				31 (27.7)						
**Item 2: “I have obtained … relevant information”**					2.4 (0.9)				2.9 (1.3)	.20		
	(1) All	3 (17.6)				17 (15.2)						
	(2)	5 (29.4)				33 (29.5)						
	(3)	8 (47.1)				23 (20.5)						
	(4)	1 (5.9)				26 (23.2)						
	(5) None	0 (0)				13 (11.6)						
**Item 3: “I could provide … information to patient”**					2.4 (1.2)				3.0 (1.2)	.14		
	(1) All	5 (29.4)				19 (17.4)						
	(2)	4 (23.5)				31 (28.4)						
	(3)	5 (29.4)				16 (14.7)						
	(4)	2 (11.8)				21 (19.3)						
	(5) None	1 (5.9)				22 (20.2)						

^a^Mann-Whitney *U* test.

### Recruitment

Recruitment of cases was carried out by 4 EMS stations in the German Federal State of Lower Saxony. Three of the rescue stations were located in rural areas with relatively long distances to the next hospital. Two of the stations serve a frequented motorway with international transit traffic. Notably, one station was in an urban setting and provided EMS to a reception center for refugees and asylum seekers. Recruitment took place between March 24, 2019, and November 15, 2021, and the app was introduced on December 15, 2019.

### Inclusion and Exclusion Criteria

Inclusion criteria for the intervention group were as follows: (1) non–German-speaking patients or LGP patients of all ages who speak one of the languages supported by the app; (2) patients responsive to the use of the app; and (3) presence of a language barrier and thus a need for language interpretation. Exclusion criteria for the intervention group were as follows: (1) emergency situation, where the use of the app would not be responsible, for example, situations where paramedics put themselves in danger, an emergency that requires immediate action (eg, cardiopulmonary resuscitation), or patient transport must take place without any delay; (2) patient declines to interact with the app; (3) patients speak a language that paramedics speak fluently (ie, absence of a language barrier); and (4) no provision of prehospital emergency care (eg, hospital to hospital transport).

Inclusion criterion for control group 2 was German-speaking patients of all ages. The exclusion criterion was no provision of prehospital emergency care (eg, hospital-to-hospital transport).

Inclusion criteria for control group 1 were as follows: (1) non–German-speaking patients or LGP patients of all ages and (2) presence of a language barrier and thus a need for language interpretation. Exclusion criteria for control group 1 were as follows: (1) patients speak a language that paramedics speak fluently (ie, absence of a language barrier) and (2) no provision of prehospital emergency care (eg, hospital-to-hospital transport). By comparing data on the intervention group and on control group 1, the immediate effect of the tool on communication and information gathering with non–German-speaking patients could be analyzed.

### Statistical Analyses

To describe the intervention and the 2 control groups, absolute and relative frequencies as well as mean values and SDs were used. Chi-square and Fisher exact tests were used to test for independence between categorical variables. Mann-Whitney *U* and Kruskal-Wallis were used for testing metric and ordinal variables. In addition to the bivariate statistical test, a linear regression model was applied between the intervention group and the control group 1 to assess the impact of the app on perceived communication while controlling for patients’ age, gender, and GCS. Additional sensitivity analyses were conducted with subsamples of control group 1. In 1 refined sample, patients who did not speak one of the languages supported by the app were excluded. For another sensitivity analysis, patients were additionally excluded from control group 1 if they were recruited after the app was implemented. These excluded patients may represent severe cases, where paramedics decided not to use the app as it would endanger themselves or the patient. For all analyses, *P* values of <.05 were regarded as significant. All analyses were performed using SPSS (version 27, IBM Corp).

### Research Ethics

The study received approval from the responsible research ethics board of the University Medical Center Göttingen (ethics approval number 9/9/18) and was registered in the German Clinical Trial Register (DRKS00016719). To participate in the study, paramedics needed to declare informed consent. No written consent was obtained from the patients. However, if patients rejected to communicate using the app, paramedics were instructed to abort the use. Written informed consent from patients was waived by decision of the research ethics board.

## Results

### Sample Characteristics

**Figure 3:**
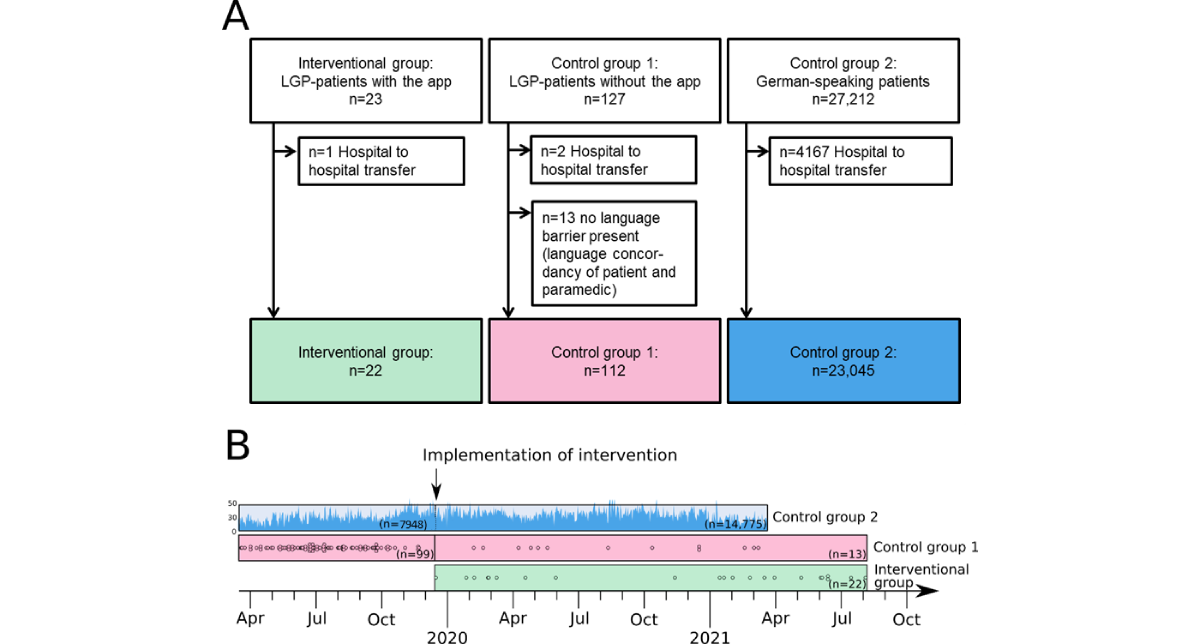
Study schema. (A) Flowchart of participant inclusion. (B) Timeframe of participant inclusion before and after app implementation. Each data point represents an included patient; for control group 2, included patients are shown as a frequency polygon, indicating recruited German-speaking patients on a daily basis (missing date in control group 2, n=322). LGP: limited German language proficiency.

A total of 22, 112, and 23,045 patients were recruited in intervention group, control group 1, and control group 2, respectively. Patient inclusion is depicted in Figure 3A. Recruitment of control group 1 mainly took place before the implementation of the intervention (Figure 3B). During the time span when the app was available, in 62.9% (n=22) out of the total of 35 recruited LGP patients the app was used. Reasons not to use the app in 13 cases included 4 patients who did not speak any of the languages supported by the app, 1 patient who declined to interact with the app, and 8 rescue missions with circumstances that made paramedic take the decision not to use the app (immediate treatment or transport needed).

LGP patients in both intervention and control group 1 were more than 2 decades younger than German-speaking patients (*P*<.001). Female patients were slightly but not significantly overrepresented in control group 1 (*P*=.23) and underrepresented in the intervention group (*P*=.01) when compared to German-speaking patients. Intervention group and control group 1 only differed in terms of patient’s sex significantly. The proportions of spoken languages in intervention and control group 1 were almost similar: Polish (n=6, 27.3% in the intervention group vs n=20, 17.9% in control group 1), Russian (n=3, 13.6% vs n=11, 9.8%), Arabic (n=2, 9.1% vs n=19, 17.0%), and Turkish (n=2, 9.1% vs n=10, 8.9%). Table S2 in Multimedia Appendix 1 shows the 23 languages spoken by patients in intervention and control group 1. Of those 99 patients included in control group 1 (before the implementation of the app), 81 spoke one of the languages supported by the app. Further characteristics of enrolled patients are shown in Tables 2 and 3.

**Table 2:** Characteristics of patients’ demographics and rescue missions.

Characteristics			Intervention group (LGP^a^ patients with the app; n=22)		Control group 1 (LGP patients without the app; n=112)		Control group 2 (German-speaking patients; n=23,045)		*P* value^b^	
**Sex^c^, n (%)**										.006
	Male	43 (45.3)		16 (80)		11,515 (51.5)				
	Female	52 (54.7)		4 (20)		10,827 (48.4)				
	Unclear	0 (0)		0 (0)		21 (0.1)				
Age^c^ (years), mean (SD)			37.0 (19.7)		41.4 (23.4)		63.1 (23.9)		.59^d^	
Children (age <18 years), n (%)			2 (9.1)		12 (10.7)		1248 (5.4)		>.99	
Emergency physicians present, n (%)			5 (25)		18 (23.7)		5223 (29)		>.99	
Patient rejected care, n (%)			2 (9.1)		2 (1.8)		1024 (4.4)		.13	

^a^LGP: limited German language proficiency.

^b^Fisher exact test intervention group versus control group 1, if not otherwise stated.

^c^Missing sex n=701, missing age n=465.

^d^Mann-Whitney *U* test intervention group versus control group 1.

**Table 3:** Characteristics of patients’ initial assessments and preliminary diagnoses.

Characteristics		Intervention group (LGP^a^ patients with the app; n=22)	Control group 1 (LGP patients without the app; n=112)	Control group 2 (German-speaking patients; n=23,045)	*P* value^b^
**Initial assessment**					
	Glasgow Coma Scale^c^, mean (SD)	14.8 (0.5)	14.5 (2.0)	14.1 (2.7)	.82^d^
	Psychiatric symptoms, n (%)	2 (14.3)	9 (11)	2605 (14.9)	.66
**Preliminary diagnosis, n (%)**					
	Neurological disorders	2 (9.1)	6 (5.4)	2322 (10.1)	.62
	Cardiovascular disorders	1 (4.5)	19 (17)	5701 (24.7)	.20
	Respiratory disorders	0 (0)	9 (8)	2141 (9.3)	.36
	Metabolic disorders	1 (4.5)	3 (2.7)	1389 (6)	.52
	Psychiatric disorders	1 (4.5)	7 (6.3)	1835 (8)	>.99
	Abdominal disorders	4 (18.2)	13 (11.6)	2162 (9.4)	.48
	Gynecological and obstetric disorders	2 (9.1)	7 (6.3)	240 (1)	.64
	Other disorder	3 (13.6)	7 (6.3)	2040 (8.9)	.21
**Injury, n (%)**					
	None	21 (95.5)	93 (83)	19,807 (85.9)	.37
	Slight	1 (4.5)	19 (17)	3238 (14.1)	.20
	Moderate	1 (4.5)	2 (1.8)	1577 (6.8)	.42
	Severe	0 (0)	0 (0)	223 (1)	N/A^e^

^a^LGP: Limited German language proficiency.

^b^Fisher exact test intervention group versus control group 1 if not otherwise stated.

^c^Missing GCS, n=666.

^d^Mann-Whitney *U* test intervention group versus control group 1.

^e^N/A: not applicable.

### Impact of the App on Perceived Quality of Communication

Perceived quality of communication was rated for each rescue mission with LGP patients by paramedics with 3 questions on a 5-point Likert scale (Table 1). The intervention group showed on average a 0.7-point better rating concerning perceived overall communication (item 1), 0.5-point better rating concerning obtaining information from patients (item 2), and 0.6-point better rating concerning providing information to patients (item 3). However, only the results of the first were statistically significant (*P*=.03). In none of the emergency cases of the intervention group, the communication was perceived as “very difficult,” compared to 27.7% (n=31) of the cases in control group 1. In none of the cases with app usage but in 11.6% (n=13) of cases of the control group 1, it was impossible to obtain at least some of the relevant information.

A general linear model controlling for patients’ age, sex, and GCS revealed that the perceived overall quality of communication was associated with an increase of 0.9 points (95% CI 0.2-1.6, *P*=.01) on the Likert scale when the app was used compared to nonapp usage. Items on obtained information or provided information did not show a significant change in the multivariable model if the app was used. Sensitivity analyses with a refined control group 1, excluding patients who did not speak a language that was supported by the app (model B) and excluding patients who were recruited after the implementation of the app (model C), confirmed the results. Respective regression tables can be found in Table S1 in Multimedia Appendix 1.

### Impact of the App on On-Scene Time

**Figure 4:**
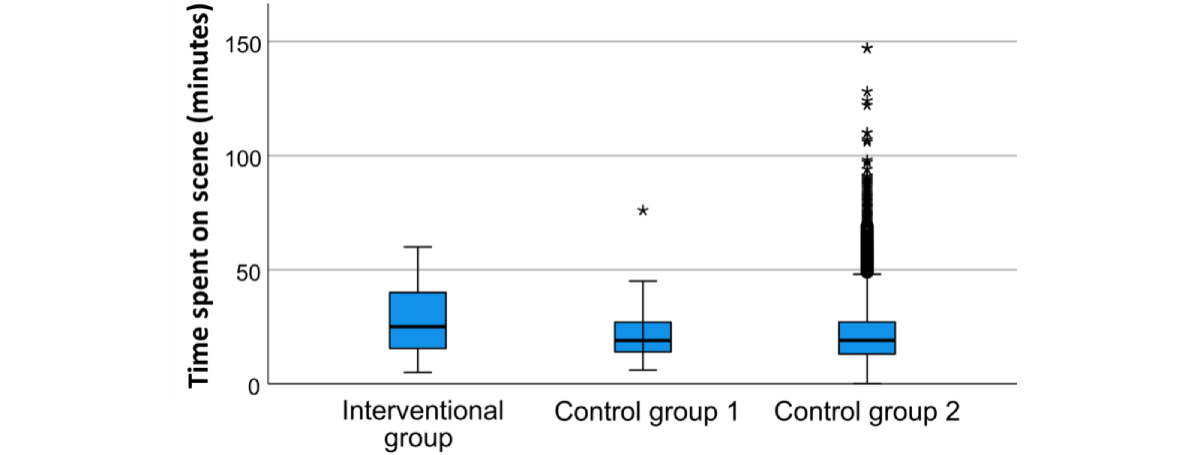
Time spent at the emergency scene.

Paramedics spent, on average, 6.5 minutes and 6.9 minutes longer on the emergency scene when the app was used compared to German-speaking and LGP patients, respectively (Figure 4). A Kruskal-Wallis test did not confirm the significance of these differences (*P*=.24).

## Discussion

### Overview

This study showed that the use of an app designed to overcome language barriers in EMS is associated with an improvement in the perceived quality of communication with foreign language–speaking patients. In a multivariable model that adjusted for patient age, sex, and disease severity, the improvement was 0.9 points on a 5-point Likert scale. Of note, in none of the cases where the app was used, paramedics rated communication as “very difficult” or stated that they had not received any information, whereas this is true for 27.7% (n=31) and 11.6% (n=13) of cases, respectively, in the comparison group with LGP patients. The items on information exchange indicated improvement through app use, but these differences were not statistically significant.

On average, the time that paramedics spent at the emergency scene was 6-7 minutes longer when the app was used compared to German-speaking patients or LGP patients treated without an app. While this difference was not significant, it suggests a tendency that the use of the app may increase on-scene time. In a previous study, it was shown that paramedics who have used the app in patient care perceive it as less complex and feel more confident about using the app [22]. Other studies suggest that on-scene time is reduced with patients with limited proficiency in the locally spoken language [25], potentially due to less time spent communicating to patients. Fixed-phrase translation apps for EMS showed to have good usability [22] and were preferred over direct translation devices such as Google Translate (Alphabet Inc) by foreign language speakers [26]. To the authors’ knowledge, this is the first study that assessed the impact of such an app on the quality of communication in a real-life setting. Our findings suggest that the use of the developed app may considerably improve communication with LGP patients in situations that paramedics often describe as frustrating [8]. Although these are the first promising results, follow-up studies with a more rigorous study design are needed to assess whether translation apps affect relevant clinical outcomes positively. Other studies highlighted that migrant patients are less often satisfied with emergency care and that EMS lack cultural sensitivity [27,28]. Patient-centered outcomes and patients’ satisfaction were not considered in this study, and measuring paramedics’ perceptions is prone to potential bias. Therefore, further research should take into account patients’ perspectives, for example, if the use of the app contributes to a more positive experience of the rescue mission, the influence of the app on the rescue mission other than on-scene time, for example, documentation quality, and the subsequent treatment in the hospital.

### Limitations

The main limitations of this study are the small sample size of the intervention group, the subsequent low statistical power, and limited generalizability. The original study design envisioned considerably more patients for the intervention group. The recruitment of control group 1 was quite successful, but due to the COVID-19 pandemic and the subsequent lockdowns during the recruitment phase, EMS usage [29] and emergency admissions at hospitals dropped considerably [30,31]. COVID-19–related travel restrictions resulted in a reduction in tourism, freight-transport, seasonal labor market, immigration, and long-distance travel. Thus, the number of eligible LGP patients dropped considerably and led to underrecruitment. Furthermore, hygiene measures, such as protective clothing, may have made it difficult to use the app. On-site examinations by paramedics were minimized and postponed to the hospital, rendering communication less important. Providing emergency care during the COVID-19 pandemic came with an extraordinary burden for health care workers. The massive increase in workload among paramedics [32,33] might have contributed to a lower response rate of questionnaires. As LGP patients were identified by these questionnaires, a number of cases were probably missed. It could also be the case that questionnaires were not filled out if communication turned out to be sufficient by using a third language.

The recruitment of control group 1 started more than 8 months earlier than the intervention group. We did this on purpose to have a broad LGP control sample where paramedics’ ratings on communication are not influenced by knowing about the app’s features. In addition, after the introduction of the app, LGP patients with whom the app was not used (eg, when patients did not speak one of the languages supported by the app) would be assigned to the control group, which would likely have introduced a selection bias. This was controlled with the use of sensitivity analyses.

Potential differences in outcomes between the intervention group and control group 1 may be due to other reasons, such as attempting to keep face-to-face interaction to a minimum due to the COVID-19 pandemic. As this was an unblinded nonrandomized trial, there are other potential sources of bias, for example, detection bias.

Other limitations include the small number of recruitment centers. The rather broad exclusion criteria, and especially that paramedics should not use the app in a rescue operation is perceived as “inappropriate” or “irresponsible” according to paramedics’ judgement, may have introduced a selection bias. However, this reflects a real-life situation.

### Conclusions

The difficulties and potentially dangerous consequences that may arise due to language barriers in EMS have to be tackled. Digital solutions, such as our app that helps paramedics to communicate with foreign-language patients, might be one way to improve care for these patients. The results make us feel positive that our tool contributes to a safer and more pleasant provision of paramedic care for foreign-language patients.

## Appendix

Multimedia Appendix 1 Regression and language spoken by patient tables.

## Abbreviations

EMS: emergency medical services
GCS: Glasgow Coma Scale
LGP: limited German language proficiency

## Data Availability

The data sets generated and analyzed during this study are not publicly available due to the decision of the responsible research ethics board but are available from the authors on reasonable request within a data-sharing agreement.
