# Correction: Overcoming Language Barriers in Paramedic Care With an App Designed to Improve Communication With Foreign-Language Patients: Nonrandomized Controlled Pilot Study

**DOI:** 10.2196/83353

**Published:** 2025-10-21

**Authors:** Frank Müller, Dominik Schröder, Eva Maria Noack

**Affiliations:** 1 Department of General Practice University Medical Center Göttingen Göttingen Germany

In “Overcoming Language Barriers in Paramedic Care With an App Designed to Improve Communication With Foreign-Language Patients: Nonrandomized Controlled Pilot Study” [1] the authors made the following correction.

The first two sentences in the Results section of the Abstract were originally published as:

A total of 22 LGP patients were recruited into the interventional group and 122 into control group 1. The control group 2 included 27,212 German-speaking patients.

They have now been corrected to:

A total of 22 LGP patients were recruited into the interventional group and 112 into control group 1. The control group 2 included 23,045 German-speaking patients.

The correction will appear in the online version of the paper on the JMIR Publications website, together with the publication of this correction notice. Because this was made after submission to PubMed, PubMed Central, and other full-text repositories, the corrected article has also been resubmitted to those repositories.
