# Supplementary material for: Overcoming Language Barriers in Paramedic Care With an App Designed to Improve Communication With Foreign-Language Patients: Nonrandomized Controlled Pilot Study
Source: JMIR Form Res. 2023 Mar 23;7:e43255. doi: 10.2196/43255 (PMC10131716; doi:10.2196/43255)
Supplement: Multimedia Appendix 1 [file formative_v7i1e43255_app1.docx]

**Multimedia Appendix 1**

Table S1: General linear model adjusting for Sex, Age, and GCS.

| **Item 1: “The overall communication with the patient was…”** | Model A (n=106) | | Model B (n=88) | | Model C (n=79) | |
| --- | --- | --- | --- | --- | --- | --- |
|  | **B (95 % CI)** | ***P*** | **B (95 % CI)** | ***P*** | **B (95 % CI)** | ***P*** |
| App utilized | -0.89 (-1.56 - -0.21) | .010 | -0.81 (-1.51 - -0.11) | .024 | -0.76 (-1.46 - -0.06) | .033 |
| Female sex | 0.05 (-0.39 - 0.48) | .835 | -0.05 (-0.53 - 0.44) | .855 | -0.08 (-0.58 - 0.43) | .760 |
| Age (continous) | 0.00 (-0.01 - 0.01) | .765 | 0.00 (-0.01 - 0.01) | .579 | 0.00 (-0.01 - 0.01) | .997 |
| GCS (continous) | 0.02 (-0.09 - 0.13) | .761 | 0.08 (-0.06 - 0.22) | .262 | 0.10 (-0.04 - 0.24) | .150 |
|  |  |  |  |  |  |  |
| **Item 2: “I have obtained … relevant information”** | Model A (n=106) | | Model B (n=88) | | Model C (n=79) | |
|  | **B (95 % CI)** | ***P*** | **B (95 % CI)** | ***P*** | **B (95 % CI)** | ***P*** |
| App utilized | -0.37 (-1.09 - 0.34) | .304 | -0.3 (-1.02 - 0.42) | .414 | -0.31 (-1.03 - 0.41) | .403 |
| Female sex | 0.14 (-0.32 - 0.59) | .549 | -0.02 (-0.51 - 0.48) | .941 | -0.13 (-0.65 - 0.39) | .634 |
| Age (continous) | 0.00 (-0.01 - 0.01) | .721 | 0.00 (-0.01 - 0.01) | .786 | 0.00 (-0.02 - 0.01) | .452 |
| GCS (continous) | -0.06 (-0.17 - 0.06) | .345 | -0.04 (-0.18 - 0.1) | .566 | -0.02 (-0.16 - 0.12) | .797 |
|  |  |  |  |  |  |  |
| **Item 3: “I could provide … information to patient”** | Model A (n=103) | | Model B (n=85) | | Model C (n=76) | |
|  | **B (95 % CI)** | ***P*** | **B (95 % CI)** | ***P*** | **B (95 % CI)** | ***P*** |
| App utilized | -0.23 (-1.02 - 0.55) | .557 | -0.15 (-0.92 - 0.63) | .710 | -0.18 (-0.97 - 0.61) | .651 |
| Female sex | 0.37 (-0.13 - 0.87) | .150 | 0.5 (-0.05 - 1.04) | .076 | 0.55 (-0.04 - 1.13) | .066 |
| Age (continous) | 0.00 (-0.01 - 0.01) | .527 | 0.01 (0.00 - 0.02) | .201 | 0.01 (0.00 - 0.02) | .139 |
| GCS (continous) | -0.23 (-0.39 - -0.08) | .004 | -0.33 (-0.56 - -0.11) | .003 | -0.35 (-0.58 - -0.11) | .004 |

Reference categories: Male sex, app not utilized. Model A: comparison with whole control group 1 (n=112), Model B: control group 1 with participants speaking a language supported by the app (n=90), Model C: control group 1 with participants speaking a language supported by the app AND recruited before the implementation of app (15 Dec 2019) (n=81).

Table S2: Languages spoken by patients in intervention and control group 1.

|  | Intervention group  (LGP patients with app) (*n* = 22) | | Control group 1 (LGP patients without app) (*n*=112) | |
| --- | --- | --- | --- | --- |
| Language | n | % | n | % |
| Albanian | 0 | 0.0 | 2 | 1.8 |
| **Arabic** | 2 | 9.1 | 19 | 17.0 |
| **Bosnian or Croatian** | 0 | 0.0 | 2 | 1.8 |
| Bulgarisch | 0 | 0.0 | 5 | 4.5 |
| **Dari (Persian)** | 0 | 0.0 | 2 | 1.8 |
| **English** | 1 | 4.5 | 1 | 0.9 |
| **Farsi (Persian)** | 0 | 0.0 | 3 | 2.7 |
| **French** | 0 | 0.0 | 4 | 3.6 |
| Georgian | 0 | 0.0 | 1 | 0.9 |
| Greek | 0 | 0.0 | 1 | 0.9 |
| **Italian** | 0 | 0.0 | 1 | 0.9 |
| **Kurdish** | 1 | 4.5 | 7 | 6.3 |
| **Lithuanian** | 1 | 4.5 | 2 | 1.8 |
| Montenegrin | 0 | 0.0 | 1 | 0.9 |
| Nigerian native language | 0 | 0.0 | 2 | 1.8 |
| **Polish** | 6 | 27.3 | 20 | 17.9 |
| Romanian | 0 | 0.0 | 4 | 3.6 |
| **Russian** | 3 | 13.6 | 11 | 9.8 |
| Slovakian | 1 | 4.5 | 0 | 0.0 |
| Somali | 2 | 9.1 | 1 | 0.9 |
| **Spanish** | 0 | 0.0 | 3 | 2.7 |
| **Turkish** | 2 | 9.1 | 10 | 8.9 |
| Ukrainian | 0 | 0.0 | 1 | 0.9 |
| unknown | 2 | 9.1 | 5 | 4.5 |
| other | 1 | 4.5 | 4 | 3.6 |

*In bold: languages supported by the app. Reference categories. Three languages supported by the app (Pashto (Afghani), Czech and Serbian) were not spoken by any of the recruited patients.
